# Sex influences the brain functional connectivity correlates of originality

**DOI:** 10.1038/s41598-021-02674-5

**Published:** 2021-12-02

**Authors:** Richard B. Silberstein, David A. Camfield

**Affiliations:** 1 Centre for Human Psychopharmacology, Swinburne University, Level 3, Building B, 192 Burwood Road, Hawthorn, VIC 3122 Australia; 2 Neuro-Insight Pty Ltd, Melbourne, 3122 Australia

**Keywords:** Neuroscience, Psychology

## Abstract

Creative cognition is thought to involve two processes, the creation of new ideas and the selection and retention of suitable new ideas. Neuroimaging studies suggest that the Default Mode Network contributes to the creation of new ideas while left inferior frontal and parieto-temporal cortical networks mediate the selection/retention process. Higher levels of activity in the selection/retention have been shown to be associated with stricter criteria for selection and hence the expression of fewer novel ideas. In this study, we examined the brain functional connectivity correlates of an originality score while 27 males and 27 females performed a low and a high demand visual vigilance task. Brain functional connectivity was estimated from the steady state visual evoked potential event related partial coherence. In the male group, we observed a hypothesized left frontal functional connectivity that was negatively correlated with originality in both tasks. By contrast, in the female group no significant correlation between functional connectivity and originality was observed in either task. We interpret the findings to suggest that males and females engaged different functional networks when performing the vigilance tasks. We conclude with a consideration of the possible risks when data pooling across sex in studies of higher cortical function.

## Introduction

Intellectual creativity or the process of creating new and useful ideas is now generally considered to involve two distinct unconscious processes. One of these creates numerous new ideas through novel combinations of pre-existing ideas while the other evaluates and judges the fitness or quality of these ideas before the one judged most fit emerges in consciousness. A widely quoted model of this process is termed the ‘Blind Variation Selective Retention’ model of creativity^1^. In the Blind Variation Selective Retention model, it is assumed that the process of creativity involves the generation of a numerous original ideas that are based on novel variations of the relationships between pre-existing ideas (Blind Variation) and a process that evaluates and selectively retains only the original idea deemed most satisfactory. More recently, studies have suggested that a specific cortical network, known as the Default Mode Network (DMN) may mediate the principal role in the generation of new ideas, irrespective of their suitability while a ‘cognitive control’ network centred on the left Inferior Frontal Gyrus (left IFG) and the left parieto temporal region is principally responsible for the process of evaluation and selective retention of novel ideas^2–5^.

In an earlier study examining the brain functional connectivity correlates of the *Abbreviated Torrance Test for Adults* (ATTA)^6^ creativity score while participants performed the AX version of the continuous performance task (CPT-AX) we observed a parieto-frontal FC component that exhibited DMN like behaviour^7^. We found that this component was positively correlated with the ATTA creativity score in both the male and female groups although we observed sex differences in the hemispheric asymmetry and timing of these FC components^7^. Our observation of sex differences in the FC correlates of the ATTA creativity score was also consistent with other studies examining sex differences in the brain functional and structural correlates of creativity^8–12^.

While the DMN has been identified as the principal network mediating the generation of new ideas, cognitive control networks, located in the left inferior frontal gyrus (left IFG) and left parieto-temporal cortex (left PTC) are thought to determine the selection and retention of original ideas based on levels of originality^13–22^.

The participation of the left IFG in the selection and retention of original ideas as opposed to the creation of new ideas was supported by fMRI studies where participants were required to both generate novel ideas and also evaluate the novelty of ideas generated by other participants^19^. Higher levels of left IFG were found to be associated with more conservative criteria for acceptance of new ideas and hence a reduction in the number of novel ideas produced while lower levels of left IFG activity were associated with an increased number of novel ideas. In other words, higher left IFG activity is associated with a reduced production of new ideas and hence a lower originality score^17,19,23^.

Brain lesion studies also point to a selection and retention role for the left parieto-temporal region. In a study examining the relationship between the locations and size of localized brain lesions and originality, it was reported that left (but not right) parieto-temporal lesions were associated with increased originality^24^.

In the current study, we examined the relationship between a measure of originality and brain FC while participants performed a low demand visual vigilance task comprising a repeated presentation of the first 5 letters of the alphabet (ABC task) and a more demanding AX version of the Continuous Performance Task (CPT-AX task). While these tasks in themselves do not engage creative cognition, our earlier findings of FC components that are correlated with the creativity score while performing these tasks suggest that that they may engage brain functional networks that also mediate creative cognition^7^.

Brain functional connectivity (FC) was estimated using an Event Related Partial Coherence methodology that we have previously used to examine cognitive task related changes in brain FC as well as the FC correlates of cognitive task performance^7,25–27^.

Considering the reviewed evidence pointing to increased left IFG activity and left temporoparietal activity being associated with more conservative or restrictive criteria for judging novel ideas as appropriate and hence the reduced production of original ideas, we hypothesize that FC components centred on the left frontal and left temporo-parietal scalp sites will be negatively correlated with the originality. Furthermore, we hypothesize this negative correlation will apply in both the male and female groups during the performance of both the low demand ABC task and the more demanding CPT-AX task.

## Results

### Abbreviated torrance test for adults (ATTA) results

Means and standard deviations of age, IQ and ATTA scaled scores for both groups are listed in Table 1. The individual originality measure (Or) that was correlated with functional connectivity (FC) was based on the ATTA Originality Score (OS) divided by the Fluency Score (FS), ie Or = OS/FS. The Or is described in more detail in the Materials sub-section of the Methods section.

**Table 1:** Means and standard deviations for demographic and ATTA scores.

*N*	Male	Female	Unpaired t-test,
	27	27	2-tail, df = 52
Age^†^	27.0 (6.8)	28.9 (5.0)	*p* = 0.23
WASI IQ	113.1 (10.3)	112.2 (4.7)	*p* = 0.69
**ATTA**			
Originality (OS)	16.5 (2.0)	17.0 (1.8)	*p* = 0.36
Fluency (FS)	15.7 (1.9)	16.3 (2.4)	*p* = 0.36
Elaboration	14.9 (1.5)	15.4 (2.2)	*p* = 0.32
Flexibility	15.8 (2.1)	15.8 (2.2)	*p* = 1.00
Creativity	71.7 (6.5)	73.9 (6.8)	*p* = 0.21
Or (OS/FS)	1.08 (0.24)	1.06 (0.15)	*p* = 0.79

Mean participant age, IQ and the sub-scores derived from the Abbreviated Torrance Test for Adults (ATTA). The originality score used in this study (Or) is the ATTA Originality score divided by the Fluence score. Other ATTA scores such as Elaboration, Flexibility and Creativity were not used in this study and are provided for the sake of completeness.

No statistically significant sex differences were observed in any of the ATTA parameters or Or. Neither of the CPT-AX performance measures, accuracy and reaction time were correlated with Or in either group. Details are provided in Supplementary Information. Performance information on the low demand ABC task is not considered relevant as the accuracy was 100% in all subjects and the onset time of the target letter was completely predictable.

### Brain functional connectivity correlates of originality during performance of the ABC and CPT-AX tasks

**Figure 1:**
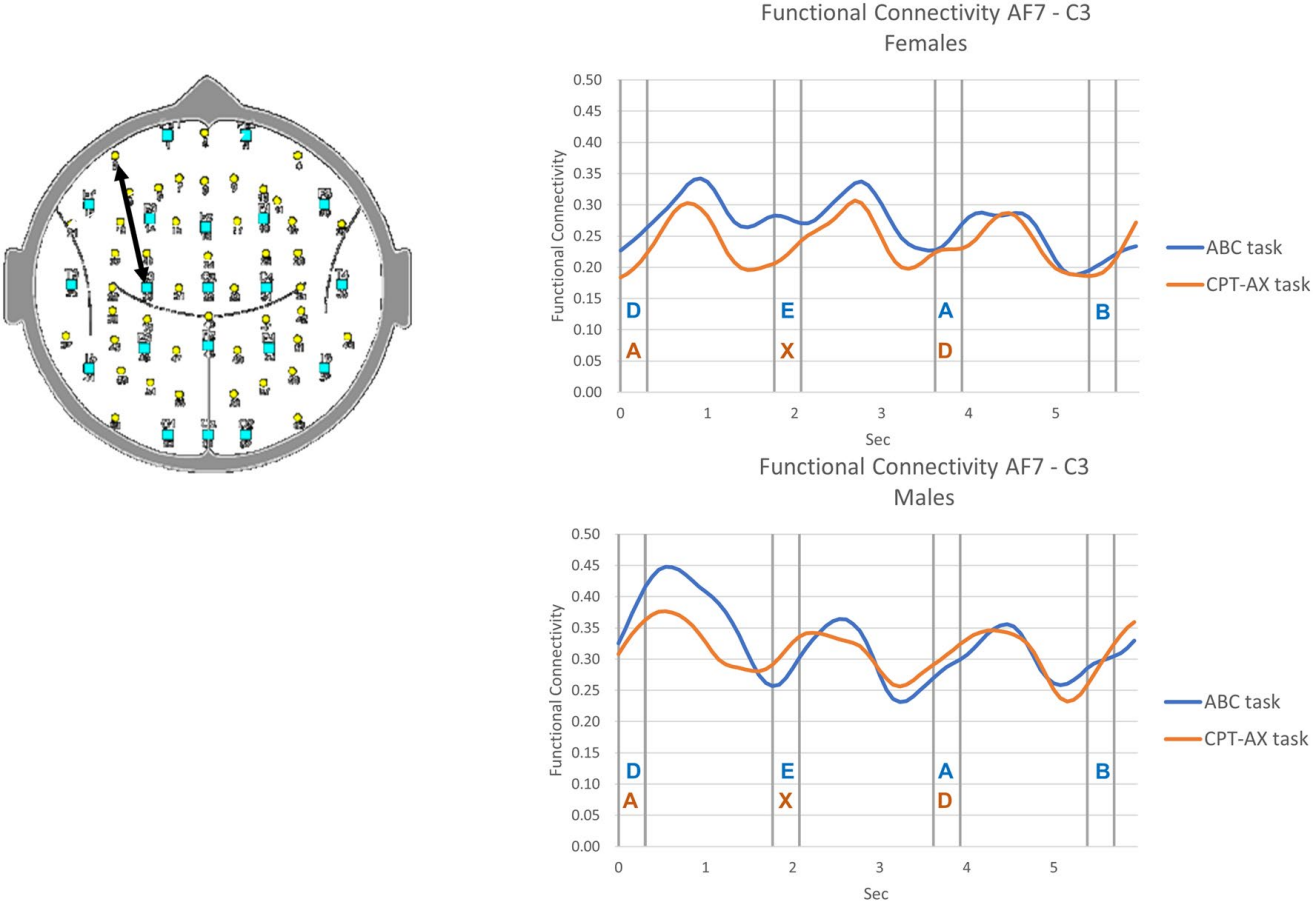
Example of functional connectivity (FC) between left frontal sites (AF7 – C3) during performance of the ABC task (blue trace) and CPT-AX task (red trace). Presentation of the vigilance task stimuli were associated with variations in FC. The horizontal line represents the average value of FC over the 6 s epoch.

**Figure 2:**
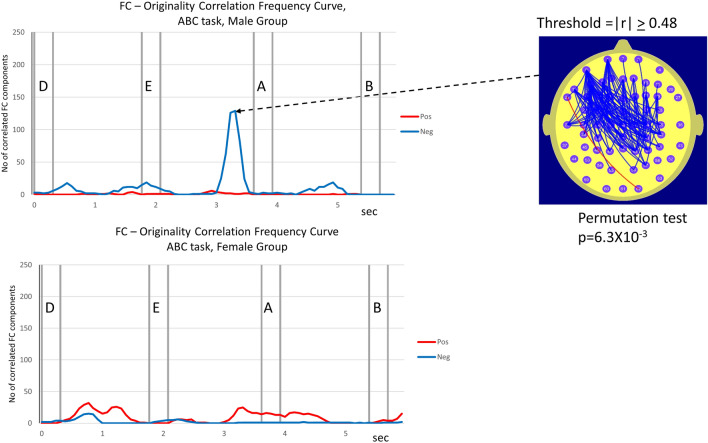
Number and location of electrode pairs where FC was correlated with the Originality score (Or) at the |r|> 0.48 level while the males (top trace) and the females (bottom trace) performed the ABC low demand vigilance task. In the graphs or *correlation frequency curves*, the blue trace illustrates the number of electrode pairs where FC is negatively correlated with Or (r ≤ -0.48) while the red trace illustrates the corresponding number that is positively correlated with Or (r ≥ 0.48). The topographic map illustrates the FC components correlated with Or (at threshold condition |r|≥ 0.48) at the point in time where the correlation frequency curve peaks. The p value listed under the map is derived using the permutation test and is the probability of observing this number of correlated FC components under the NULL hypothesis. The topographic maps are presented only if the permutation test indicates that the number of correlated FC components is statistically significant at the p ≤ 0.05 level. This level of significance was only observed in the male data. In the case of the female data, the peak of the correlation frequency curve for positive correlations peaks at the point 0.77 s into the trial corresponding to 32 FC correlated with Or at the threshold condition |r|≥ 0.48. The probability of observing this number of correlated FC components is *p* = 0.06 and thus does not satisfy the criterion for an illustrative topographic map.

The appearance of each letter in the ABC and CPT-AX tasks are associated with transient FC changes. The FC changes for one such electrode pair and for both tasks and both sexes are illustrated in Figs. 1, 2.

During the ABC task, we observe a statistically significant number of left frontal and prefrontal to central and right posterior FC components that are negatively correlated with Or only in the male data. For the male data the *correlation frequency curve* for the |r|≥ 0.48 threshold condition peaks at a value of 129 FC components approximately 400 ms prior to the appearance of the letter ‘A’. The permutation test indicates that the probability of this number of correlated FC components occurring by chance is *p* = 6.3 × 10^–3^.

By contrast, at no time during the ABC task does the permutation test indicate that the female *correlation frequency curve* reached the *p* < 0.05 level of statistical significance.

During the more demanding CPT-AX task, the male data exhibits a similar topography to that seen in the ABC task. Specifically, left frontal/prefrontal to central/parietal FC components that negatively correlated with Or. This effect was even stronger than that observed in the ABC task with the corresponding |r|≥ 0.48 threshold *correlation frequency curve* reaching 258 FC components satisfying this threshold approximately 400 ms prior to the appearance of the ‘X’. In light of the large number of FC components satisfying the |r|≥ 0.48 threshold, we also include the map illustrating the FC components satisfying the more demanding |r|≥ 0.60 threshold. The permutation test for both thresholds |r|> 0.48 and r ≥ 0.60 yields the same the same probability (*p* = 7.0 × 10^–4^) of this number of correlated FC components being observed in either of the |r| thresholds. The striking sex difference observed in the ABC findings were also apparent in the CPT-AX findings where the value of the female *correlation frequency curve* did not reach a level of statistical significance.

The statistical significance of the sex difference in the number of FC components correlated with Or was determined using a permutation test. In each task, the permutation test was applied at the points in time where the sex difference in the number of correlated FC components was a maximum. The sex difference in the number of correlated FC components was statistically significant in both tasks. The details are provided in Table 2.

**Table 2:** Number of FC components correlated with Or in female and male groups at specified points in time for ABC task and CPT-AX task.

Task	Point in time	No of correlated FC components, female group	No of correlated FC components, male group	Statistical significance of sex difference, p
ABC	3.4 s	1	129	*p* = 2.1 × 10^–3^
CPT-AX	1.5 s	2	258	*p* = 8.0 × 10^–4^

Column 2 indicates the point in time for the female-male comparison of the number of FC components correlated with Or during the ABC and CPT-AX tasks. The number of correlated FC components is listed for both females and males and the statistical significance of the sex difference in the number of correlated FC components is listed in the subsequent 3 columns. The statistical significance of the sex difference in the number of correlated FC components was determined using the described permutation test.

## Discussion

To the best of our knowledge, this is the first demonstration of a correlation between an SSVEP-ERPC measure of brain functional connectivity (FC) and originality (Or). Two major findings emerge from this study, one predicted and one not. The first is the hypothesized negative correlation between Or and left frontal FC during both tasks in the male data. The prediction of a negative correlation between these measures was based on findings briefly reviewed in the introduction. Specifically, higher activity in the left inferior frontal gyrus (IFG) and left parieto-temporal region are associated with a greater likelihood of rejecting novel ideas, thus producing fewer original ideas and hence a lower originality score^17,19,23^.

The second major finding which was not predicted is that the hypothesized correlation between Or and left frontal FC was not apparent in the female data. Our findings indicate that these correlations are strongly influenced by sex even though sex itself had no significant effect on OS, FS or Or. Given the striking sex differences in the FC findings, we commence with a discussion of the male data before a consideration of the significance of the sex differences.

The male group findings support our hypotheses in that left frontal FC components are negatively correlated with the originality score, Or in both the ABC and CPT-AX tasks. Confirmation of the hypotheses appears robust in that the negative correlation between Or and FC where |r|> 0.48 (considered a strong effect size^28^) was observed in 129 FC measurements in the ABC task and 258 FC measurements in the CPT-AX task.

Interestingly, in both tasks, these effects were observed at only one point in time. In the ABC task, the correlation frequency curve peaked after the target ‘E’ and approximately 400 ms before the letter ‘A’ that starts the next repetition of the letter sequence. In the case of the CPT-AX task, the correlation frequency curve peaked approximately 400 ms before the letter ‘X’ in the target sequence of ‘A’ followed by ‘X’. We speculate that in the male group, the functional networks mediating the (Or) originality score are briefly engaged during both tasks. Thus, even though the ABC and CPT-AX tasks may not elicit any creative cognition, we suggest that these tasks nevertheless transiently engage the left frontal and left parieto-temporal networks in a manner that is proportional to what occurs in a creative cognition task. Thus, when these left frontal and left parieto-temporal networks are engaged in the ABC and CPT-AX tasks, their level of functional connectivity will be correlated with the originality score.

In considering the question of why the correlation frequency curves peak at the specific points in time during the task, we note that both correlation frequency curve peaks coincide with what could be considered a rule-based change in the response to the letter or letters subsequently to appear. Specifically, in the ABC task, the correlation frequency curve peaks after the appearance of the target letter ‘E’ where the next four letters require no response. In the more demanding CPT-AX task, the appearance of the letter ‘A’ marks a rule-based change in that a response is required if and only if the subsequent letter appearing is ‘X’. We speculate that the timing of these peaks in the correlation frequency curves is related to the role of the left IFG in executing rule-based behaviour.

The lateral frontal and prefrontal cortex are recognized to play an essential role in the acquisition and execution of either explicit or implicit rule-based behaviour^29–32^. The role of the left IFG in the acquisition and execution of rule-based behaviour has been most clearly demonstrated in studies that require participants to learn the rules of an artificial grammar. In one study, participants were exposed to grammatically correct examples of an artificial grammar letter string over a learning period of 8 days. In a subsequent fMRI recording session where participants judged text strings as grammatically correct or not, the left IFG exhibited the most consistent activity increase associated with syntactic violation^33^.

The role of the left IFG in executing rule-based behaviour in an artificial grammar task was confirmed in a trans-cranial magnetic stimulation (TMS) study where stimulation applied to the left, but not right IFG was associated improved likelihood of correctly rejecting syntactically incorrect letter strings^34^. Similar findings were reported in a study where participants first implicitly learnt the rules of an artificial grammar while an electrical DC stimulus (DCs) was applied through an anodal (stimulatory) electrode located over Broca’s area. In a subsequent classification task, DCs but not sham DCs was associated with improved rejection of syntactically incorrect letter strings^35^.

We suggest that the role of the left IFG in executing rules-based behaviour is also consistent with our observation of the correlation between the Or score and left frontal FC. We base this suggestion on the fact that the selection-retention process in creative cognition is based on the application of implicit or explicit rules to determine the suitability of a novel idea. Thus, creative cognition is but one type of cognitive process that draws upon the neural network that mediates rules-based behaviour. While the current study tasks did not involve creative cognition, we suggest that they did engage the neural network mediating rules-based behaviour, hence the observed link between the originality score and the left frontal FC components. In summary, the male data supports the hypothesis and is consistent with other evidence pointing to the role of the left IFG and left parieto-temporal networks in mediating the selection-retention function during creative cognition.

The female group findings are in striking contrast to the male findings in that no significant correlation between FC and originality was observed. We interpret this sex difference to indicate that the males, in performing both tasks, consistently engaged cortical networks whose level of functional connectivity during the task is correlated with that occurring during a creative cognition task. By contrast, when performing the ABC and CPT-AX tasks, we speculate that females may engage other networks that do not play a role in mediating the individual originality score. Even though none of the FC components correlated with Or during both tasks, the female group performance level on this task was equivalent to the male performance. Furthermore, as illustrated in Fig. 1, both the male and female groups exhibited robust task related changes in FC.

Importantly, our data should not be inferred to suggest that female participants fail to engage the left IFG and/or left parieto-temporal networks when engaged in creative cognition. What our data does suggest is that females, as a group do not engage these networks in the vigilance tasks used in this study. Furthermore, we would predict that a female group would exhibit a comparable correlation between left frontal FC components and originality when performing a creativity judgement task of the type previously described^19,36^.

Our observations of the pronounced sex difference in these findings is consistent with a growing body of reports indicating sex differences in both the structural and functional correlates of creativity. Sex differences in white matter relative volume and connectivity correlates of creativity have been reported^10–12^ and such sex-based differences in the EEG correlates of creativity have also been reported^37–39^.

In an fMRI study, it was reported that males compared to females, exhibited higher brain activity in several regions including the left inferior frontal cortex during a divergent thinking task^9^. Sex differences in the resting state brain FC correlates of creativity as measured by a divergent thinking task have also been observed in a large study (545 females, 732 males)^12^ where a correlation between resting state FC between the medial prefrontal cortex and the left inferior frontal gyrus was observed. However, this correlation was positive for the female group and negative for the male group. In a subsequent study conducted by the same group, areas of the left parietal lobe and left precuneus were positively correlated with originality in the female group (n = 521) and negatively in the male group (n = 700)^40^.

While creative cognition may be considered a complex task drawing on a range of cognitive processes, similar sex-based differences have also been observed in a less complex task, such as long-term memory retrieval^41^. In summary, our findings of sex differences in brain FC correlates of the originality measure are not only consistent with a range of EEG and fMRI studies but also consistent with our earlier report of sex differences in the FC correlates of the ATTA creativity score^7^.

This study, like most suffers from several limitations. One of the major limitations is the relatively poor spatial resolution of the FC components. This made it difficult to relate the FC components identified in this study with specific neuroanatomical areas identified in the fMRI-based studies of originality. Another factor is the failure to consider factors that may have contributed to the lack of any significant correlation between Or and FC in the female group. One possible factor may have been greater inter-subject FC variability in the female group caused by hormonal changes associated with the menstrual cycle^42^. This factor was not considered in the design of the study.

While we consider our findings suggestive, they need to be confirmed in a larger and ideally, independent study. In considering any such replication study, it is important to note that the findings depend very much on the methodology used to determine FC. Our method using the 13 Hz SSVEP-ERPC to determine FC will be biased to cortical communication components mediated by oscillations around 13 Hz. This is important as ‘top-down’ or ‘feedback’ cortical communication is thought to be mediated by synchronous oscillations in the 10 Hz – 20 Hz range and thus our findings are preferentially sensitive to top-down processes^43^. Readers are referred to our previous publication for a more detailed discussion of this point^26^.

In broad terms, our findings are consistent with reports suggesting a role for the left IFG and left parieto-temporal regions in the selection/retention component of creative cognition. Our findings also indicate robust sex-based differences in the engagement of left frontal FC components correlated with Or during the performance of both visual vigilance tasks. Our observation of sex-based FC differences is consistent with a growing body of neuroscience research pointing to such differences at the molecular biology level as well as at the structural and functional levels^44–46^. Our findings also reinforce the need for significant caution in pooling brain functional data across sex. Where sex differences exist, pooling data across sex at best, dilutes findings and at worst, may yield irreproducible and possibly invalid findings. We recommend a conservative approach where such data pooling across sex is restricted to cases where it has been demonstrated that no significant sex-based differences are apparent in the data.

## Methods

### Participants

Fifty-four participants were enrolled in the study, comprising 27 females and 27 males. All participants gave their written informed consent to participate in the study. Mean age and IQ details are listed in Table 1. All participants were between 18 and 41 years old and were screened for the presence of pre-existing medical, neurological or psychiatric conditions, including epilepsy. Participants were recruited via advertisements placed around Swinburne University, Hawthorn, Victoria, Australia as well as through the research participant database associated with the Brain Sciences Institute. All testing and data collection was conducted at the Brain Sciences Institute, Swinburne University. The study, Project 0607/102 was approved by the Swinburne University Human Research Ethics Committee and all the methods were performed in accordance with the guidelines of the Swinburne University Human Research Ethics Committee.

### Materials

The *Abbreviated Torrance Test for Adults* (ATTA)^6^ is an abbreviated version of the Torrance Tests of Creative Thinking. It is a paper-and-pencil assessment of creative ability comprising one verbal and two figural tasks. Responses to the three tasks yield four sub-scores for abilities termed fluency, originality, elaboration and flexibility and a creativity score (CS) which is derived from the sub-scores. Full scale IQ was assessed using the *Wechsler Abbreviated Scale of Intelligence* (WASI)^47^.

In the current study, the individual measure of originality was based on the ratio of two sub-scores derived from the ATTA, the Originality sub-score and the Fluency sub-score. The Originality sub-score (OS) is based on the number novel or uncommon responses made by the participant to two verbal tasks and a figurative task. By contrast, the Fluency sub-score (FS) is based on the number of responses to the same three tasks, irrespective of how novel or uncommon the responses are. As the FS is known to contributes to the OS^48^ we controlled for FS by using the ratio OS/FS as the measure of individual originality (Or), an approach adopted by other researchers in this area^40,49^.

### Cognitive tasks

All participants performed a low demand visual vigilance task (ABC task) followed by the more demanding AX version of the Continuous Performance Task (CPT-AX) like those we have described previously^7,25,26^. This sequence was repeated so that the low demand ABC task and the CPT-AX task were each performed twice. In the low demand ABC task, participants viewed a repeated presentation of the letters A, B, C, D and E and were required to press a micro-switch on the appearance of the letter E. In the CPT-AX task participants were required to respond on the unpredictable appearance of the letter X if it had been preceded by the letter A. In all tasks, the letters remained on the screen for 300 ms and were followed by a blank screen for 1.5 s. All the letters where white and presented on a black screen. The ratio of targets to non-targets was 1:4. Both the low demand visual vigilance task and the CPT-AX task were 180 s in duration. Reaction time was recorded to an accuracy of 1 ms. For all tasks, a correct response to a target was defined as one that occurred no less than 100 ms and no more than 1.5 s after the appearance of the target (E or an X preceded by an A). Any responses outside the “correct” time intervals were defined as errors of commission, or false alarms, while failure to respond in the correct interval was defined as an error of omission.

The vigilance tasks were presented on a computer monitor. Each letter subtended a horizontal and vertical angle of approximately 1.0° when viewed by subjects from a fixed distance of 1.3 m. The stimulus used to evoke the steady state visually evoked potential (SSVEP) was a spatially diffuse 13-Hz sinusoidal flicker subtending a horizontal angle of 160° and a vertical angle of 90°, which was superimposed on the visual fields. This flicker was present throughout the task and special goggles enabled subjects to simultaneously view the cognitive task and the sinusoidal flicker. The modulation depth of the stimulus when viewed against the background was 45%.

Our choice of 13 Hz as the SSVEP stimulus frequency was based on pragmatic and theoretical grounds. Pragmatically, we have found the 13 Hz SSVEP phase and 13 Hz SSVEP partial coherence to be especially sensitive to cognitive task effects while offering a high signal to noise ratio^7,25,26^. The theoretical basis for our choice of 13 Hz as the SSVEP stimulus frequency is the recognition that the bottom-up (feed forward) and top-down (feedback) intracortical communication is mediated by synchronous oscillations at distinct frequency channels. The sensory or bottom-up communication is primarily mediated by synchronous oscillations in the EEG gamma frequency range (40 Hz and above) and to a lesser extend in the EEG theta frequency range (3 Hz – 8 Hz). By contrast, the top-down communication is mediated by synchronous oscillations in the EEG high alpha- low beta range ( 10 Hz – 20 Hz).^43^. While sensory information is processed primarily via bottom-up processes, higher cortical functions that include planning, decision-making, rules-based behaviour and presumably creative cognition are mediated by top-down processes^43^. As this study is concerned with the functional connectivity correlates of higher cortical function we consider the 13 Hz SSVEP stimulus frequency optimally located in the top-down frequency range^26^.

### Recording and processing brain electrical activity

Brain electrical activity was recorded from 64 scalp sites that included all international 10–20 positions, with additional sites located midway between 10–20 locations. The specific locations of the recording sites have been previously described^50^. The average potential of both earlobes served as a reference and a nose electrode served as a ground. Brain electrical activity was amplified and bandpass filtered (3 dB down at 0.1 Hz and 30 Hz) before digitization to 16-bit accuracy at a rate of 400 Hz. The major features of the signal processing have been described^25,51^. Briefly, the SSVEP was determined from the 13-Hz Fourier coefficients evaluated over 10 stimulus cycles at the stimulus frequency of 13 Hz, thus yielding a temporal resolution of 0.77 s. The 10-cycle evaluation period was shifted 1 stimulus cycle and the coefficients were recalculated for this overlapping period. This process was continued until the entire 180 s of activity for each task was analysed. An identical procedure was applied to data recorded from all 64 recording sites.

### Steady state visually evoked potential event-related partial coherence (SSVEP-ERPC)

For each subject, the SSVEP Event Related Partial Coherence (SSVEP-ERPC) was calculated for all 2016 pairs of electrodes averaged across all correct responses in the vigilance task. The figure of 2016 or the number of unique pairs of electrodes drawn from a total of 64 electrodes is given by the expression n*(n-1)/2 where n is the number of electrodes, that is (64*63)/2. The SSVEP-ERPC is a measure of the partial coherence between electrode pairs at the stimulus frequency eliciting the SSVEP^25,51,52^. The SSVEP-ERPC varies between 0 and 1 and like coherence, is a normalized quantity that is not determined by the SSVEP amplitude at either electrode site. Electrode pairs with high partial coherence indicate relatively stable SSVEP phase differences between electrode pairs across trials. This occurs even though SSVEP phase differences between each of the electrodes and the stimulus may be variable across trials and is equivalent to the removal of the common contribution from the SSVEP stimulus. This means that high SSVEP partial coherence between electrodes reflects a consistent synchronization between electrodes at the stimulus frequency and is not simply a consequence of two unrelated regions increasing their response to the common visual flicker.

Such synchronization reflected in the SSVEP-ERPC is thought to reflect functional connectivity between the relevant regions and as mentioned earlier, we will use the terms ‘SSVEP-ERPC’ and ‘functional connectivity’ (FC) interchangeably.

In both the ABC and CPT-AX tasks, functional connectivity was determined for a 6 s interval following the appearance of the alerting target. In the ABC task, this was the appearance of the letter ‘D’ while in the CPT-AX task it was the letter ‘A’.

For each subject, the SSVEP-ERPC was evaluated across all correct trials.

### Statistical considerations

To examine the relationship between the originality measure (Or) and brain FC the linear correlation between Or and FC were calculated for each point in time for the male and female groups. Each of these yielded 2016 time-series illustrating the correlation between FC and Originality over the 6 s epoch. To explore temporal variation in the strength of the correlation between Or and FC we determine the number of electrode pairs where the correlation between FC and Or exceeds a predetermined value at each point in time^25–27,32^.

**Figure 3:**
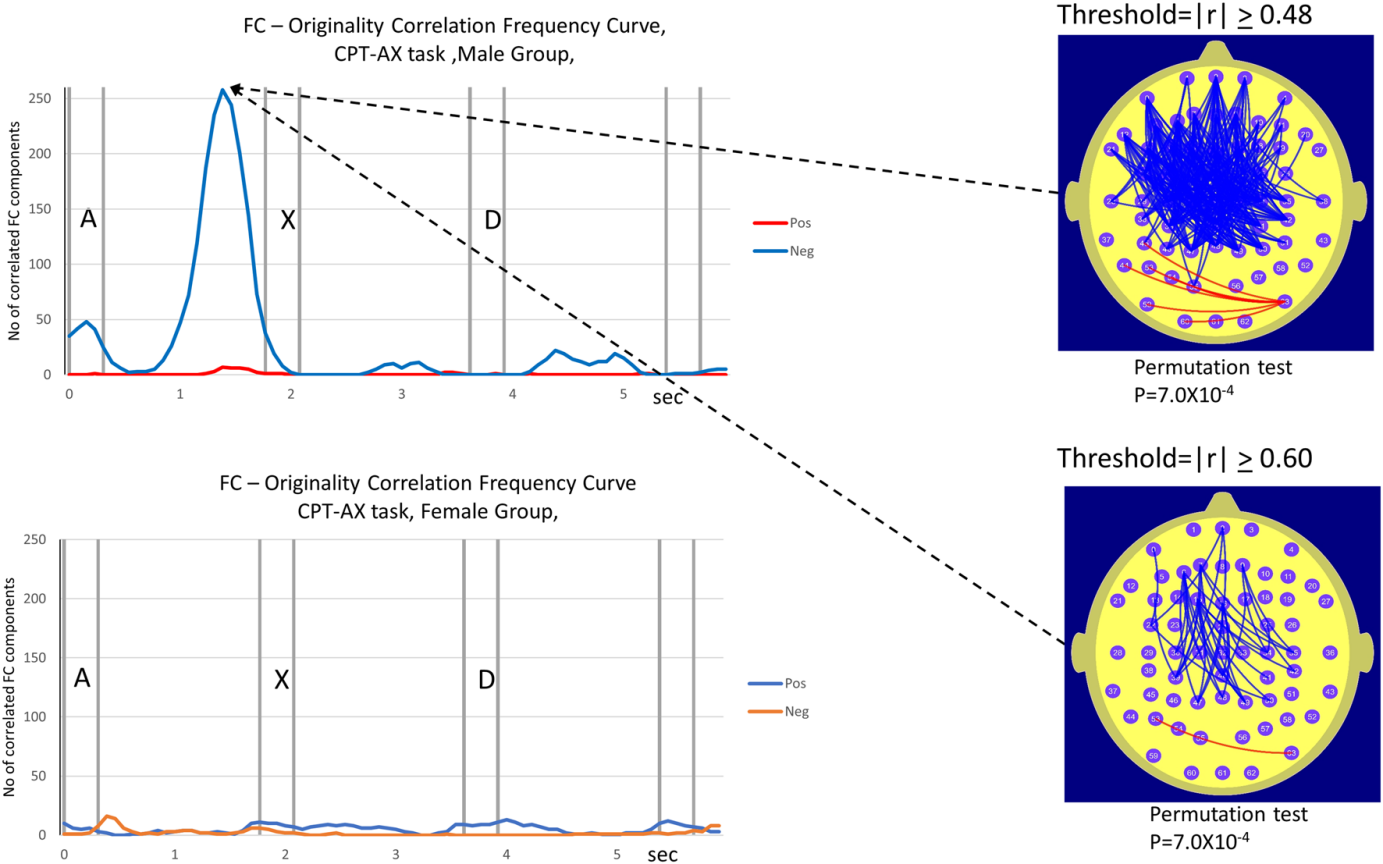
Number and location of electrode pairs where FC was correlated with the Originality score (Or) at the |r|> 0.48 while the males (top trace) and the females (bottom trace) performed the CPT-AX high demand vigilance task. The formats and conventions described in Fig. 2 also apply to this figure. Due to the large number of FC components correlated with Or at the |r|> 0.48 level in the male data, we include an additional illustration of the number of FC components correlated with Or at the more conservative level of |r|≥ 0.60.

**Figure 4:**
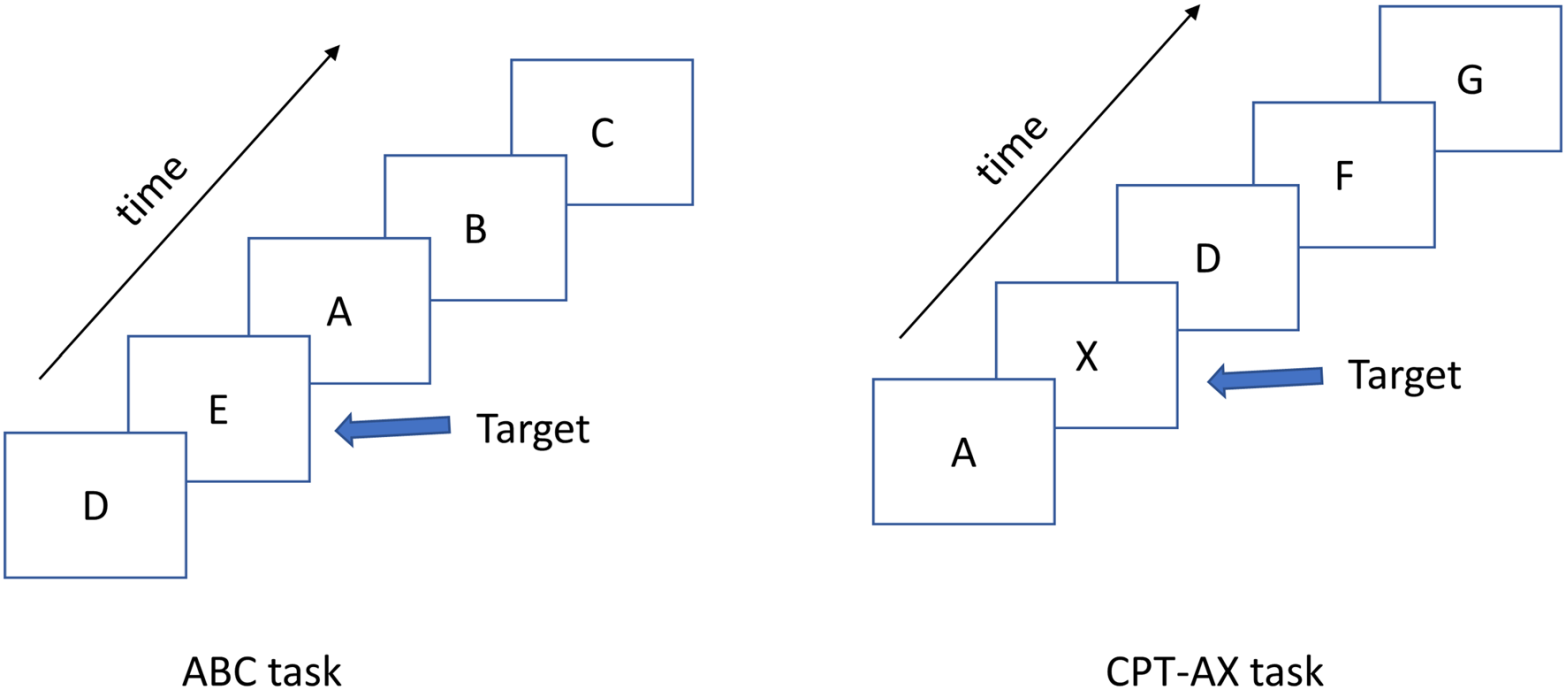
Typical sequence of letters presented in the ABC and CPT-AX tasks. In both tasks, images appeared on the screen for 300 ms and were followed by a blank screen with a duration of 1,500 ms. The ABC task presented a repeated sequence of the letters A, B, C, D, E with the requirement to press a microswitch on the appearance of the letter ‘E’. In the CPT-AX task, participants are required to press the microswitch on the appearance of the letter ‘X’ if it had been preceded by the letter ‘A’.

In the current study we determine the number of electrode pairs where the magnitude of the correlation coefficient r exceeds 0.48, (|r|≥ 0.48) a threshold value corresponding to *p* = 0.01(df = 25) at each point in time. The graphs in Figs. 3 and 4 are termed ‘*correlation frequency curves’* and comprise plots illustrating the temporal variation in the number of FC measures correlated with Or where the |r| threshold values are either met or exceeded.

We use a permutation test to determine the level of statistical significance associated with a given number of electrode pairs where the threshold value of |r| is equaled or exceeded. Although the permutation test has been described previously^25,26^, we take the opportunity to describe it here for the convenience of readers. For any given point in time in the male or female group correlation frequency curves, the number FC – Or correlations equal to or exceeding the |r| threshold is determined and designated as N_r0_. The individual Or for all participants in either the male or female groups are then randomized so that any given FC and Or are unlikely to be associated with the same individual. The number of FC – Or correlations satisfying the threshold condition is then calculated (N_ri_) and the process is repeated 10,000 times (i = 1 to 10,000). This enabled us to determine the probability of observing the N_r0_ correlations satisfying the threshold condition on the assumption that the Null hypothesis applies.

The statistical significance of the sex difference in the FC-Or correlations was evaluated using a permutation test. The statistical significance of the sex difference in the number of FC components correlated with Or at or above the |r| threshold condition was determined using a permutation test. In the case of the ABC task, we determined sex the difference in the number of correlated FC components at the point in time where the correlation frequency curve peaked in the male group. To determine the probability of this difference occurring by chance, all 27 female and 27 male data was randomly assigned to two surrogate groups and the difference in the number of correlated FC components was recalculated. This randomization process was repeated 10,000 times and the proportion of times that the proportion of randomized differences exceeding the observed one yielded the probability that our observation is consistent with the null hypothesis (no sex difference). The same process was repeated for the CPT-AX task. The main advantages of the permutation test is that it is distribution free and hence does not rely on any assumption of normality. In addition, it yields a specific probability (p) of falsely rejecting the null hypothesis.

## Supplementary Information

Supplementary Information.

## Data Availability

The dataset generated and analysed during the study described here are available from the corresponding author on reasonable request.
