# Supplementary material for: Sex influences the brain functional connectivity correlates of originality
Source: Sci Rep. 2021 Dec 2;11:23269. doi: 10.1038/s41598-021-02674-5 (PMC8640048; doi:10.1038/s41598-021-02674-5)
Supplement: Supplementary file 1 — Supplementary Information. [file 41598_2021_2674_MOESM1_ESM.docx]

**Supplementary Information**.

**Title:** ‘Sex Influences the Brain Functional Connectivity Correlates of Originality’

**Authors**: Richard B. Silberstein, David A. Camfield

| **CPT-AX task** | **Female** | **Male** | **Df, t,** | **P, Sex difference** |
| --- | --- | --- | --- | --- |
| **Accuracy %** | 95.7 SD 11.7 | 98.1 SD 3.4 | Df=52, t=1.02 | P=0.3 NS |
| **Reaction Time ms** | 501ms SD 148 | 430ms SD 88 | Df=52, t=2.14 | P=0.03 |

**Performance measures CPT-AX Task**

**Supplementary Information Table 1**. Female and male performance measures on CPT-AX task. There were no statistically significant differences in task accuracy although the male group reaction time was significantly less than female group reaction time.

**Correlation between CPT-AX performance measures and Creativity and Originality scores for male and female groups.**

|  | **Female** | | **Male** | |
| --- | --- | --- | --- | --- |
| **ATTA score** | **Accuracy %** | **Reaction Time ms** | **Accuracy %** | **Reaction Time ma** |
| **Originality, Or** | r=0.04, NS | r=o.13, NS | r=0.02, NS | r=-0.11, NS |
| **Creativity** | r=-0.15, NS | r=-0.15, NS | r=0.11, NS | r=-0.03, NS |

**Supplementary Information Table 2**. The line labelled ‘Originality’ lists the correlation between the Originality score (Or) and CPT-AX Accuracy, and CPT-AX reaction time. The next line labelled ‘Creativity’ provides the matching correlation measures for the ATTA creativity score.
